# Nutritional Sustainability Inside–Marketing Sustainability as an Inherent Ingredient

**DOI:** 10.3389/fnut.2019.00084

**Published:** 2019-06-06

**Authors:** Sabine Bornkessel, Sergiy Smetana, Volker Heinz

**Affiliations:** ^1^German Institute of Food Technologies (DIL e.V.), Quakenbrück, Germany; ^2^University of Applied Sciences, Osnabrück, Germany

**Keywords:** nutritional sustainability, ingredient branding, sustainable sourcing, communication in agrifood chains, reducing complexity

## Abstract

Current discussions about the concept of nutritional sustainability show a high complexity of this topic leading to many different definitions. Regarding communication issues of nutritional sustainability between actors of food chains this complexity should be reduced. One opportunity to tackle these challenges of reducing complexity might be the concept of ingredient branding. Therefore, the aim of this mini-review is the identification of conditions for ingredient branding application as a communication strategy for nutritional sustainability which might overcome challenges in communicating the complexity between the different stakeholders of supply chains. In doing so, the specific case of agrifood chains is discussed based on the selected characteristics of globalization, increasing consumer demands, foods incorporating credence attributes and price. Along the agrifood chain, a sourcing strategy reflecting nutritional and sustainable aspects might lead to an ingredient branding strategy implying a brand policy for a special ingredient within the final product which is an important component but cannot be clearly recognized by the user. A “nutritional sustainability inside” strategy should reflect the multifaceted information along the agrifood chain and should be based on standardized criteria for nutritional sustainability.

## Definition of Nutritional Sustainability

In recent years the importance of sustainability in agrifood chains is widely discussed in scientific literature as well as in public (1–3). In these discussions, sustainability is very often reduced solely to ecological factors (4). However, the concept of sustainability goes far beyond ecological factors including at least also economic and social factors (based on the UN Sustainable Development Goals). Concept of “sustainable diet” actively discussed for more than 40 years (5), includes not only environmental, economic and social aspects, but also ethical aspects of “healthy life for present and future generations,” “protection and respect to biodiversity and ecosystems,” “cultural acceptability,” “accessibility, economic fairness and affordability,” “nutritional adequacy, safety and health,” and “optimization of natural and human resources” (6). Such diverse and complex definition resulted in numerous approaches relying on defining specific “sustainable diets” and their role in food systems (7–10), which however did not go beyond recommendations for dietary guidelines (11). Therefore, there is a strong need to find holistic scientific foundation through future research to enhance sustainable food consumption and avoid unintended consequences of dietary guidelines based on currently limited information (12).

Moreover, different product categories require additional specific determinants, like the nutritional quality of food products in case of the agrifood sector. But only a few concepts focus on the inclusion of nutritional aspects in quantitative food sustainability models (13, 14). Assessment of separate sustainability aspects for example with life cycle assessment resulted in dilemma that favorable environmental results do not necessarily lead to a balanced diet (15). That means that a low environmental impact is not automatically connected to a high nutritional quality of a certain food (16, 17); thus, nutritional quality and environmentally favorable production can be positively as well as negatively correlated. The examples of challenges toward finding efficient solutions for the promotion of more sustainable nutritional choices show that sustainability is a very complex research area relying on multifaceted factors which cannot be easily summarized in one simple outcome. However, to improve agrifood chains and in the end consumers' decisions about sustainable nutritional food choices it is important to reduce complexity of information.

In terms of sustainability in food systems, various models define a sustainable nutrition as a form in which the raw materials of foods are produced in a sustainable way, whereas more recent studies also include the nutritional quality as a separate determinant (18–20). One of the first holistic definitions of the nutritional sustainability is delivered by Swanson with co-authors: “Nutritional sustainability is the ability of a food system to provide sufficient energy and essential nutrients required to maintain good health in a population without compromising the ability of future generations to meet their nutritional needs.” (21). Recently published study (11) provided further differentiation to the definition indicating it as “the ability of human communities (as key driving nodes) to find ways of complex food system transformation toward limited consumption of natural resources within regional or planetary boundaries while fulfilling own nutritional needs.” Accepting the definition as a guiding concept requires simultaneous consideration of ecological as well as nutritional factors in food meals and diets (11). On the one hand, the production methods of the inherent ingredient, thus the raw materials show a great impact on the overall sustainability of a product (22). On the other hand, a balanced diet is only possible if consumed food ingredients (functional properties of nutrients) can fulfill nutritional needs of a person (23) over a period of time (24). Regarding the simultaneous consideration of ecological as well as nutritional factors as an example to measure nutritional sustainability, the inherent ingredients in the products are pivotal (25, 26). Therefore, one opportunity to measure nutritional sustainability is the consideration of the inherent ingredients as building blocks of food products (27, 28). Having environmental and nutritional aspects interlinked to separate block-ingredients can lead to ingredient branding strategy as the characteristics of the inherent ingredients will define the benefits of the product and can be marketed throughout the whole value chain (29, 30). Therefore, the aim of this mini-review is to identify the conditions of ingredient branding concept application to overcome challenges in communicating the complexity of nutritional sustainability along the agrifood chain and different stakeholders and thus promote sustainable transparency. This may lead to a reduction of complexity in sustainability communication for the different stakeholders throughout the agrifood chain to improve the sustainability of the whole chain as well as better informed food choices especially at the upstream end of agrifood chain.

This paper makes a brief analysis of different approaches toward connections between “food ingredients,” “environmental impact,” and “nutritional quality.” The literature research was performed in open literature databases and search engines of “Google Scholar,” “Mendeley,” and “WorldWideScience” in 2018 and beginning 2019 using the terms: “nutrition,” “environmental impact,” “Life Cycle Assessment,” “food system,” “sustainable nutrition,” “sustainable sourcing,” “sustainable diet,” “sustainable ingredients,” “ingredient branding.” The search aimed to analyse original studies, case studies, reviews, or highlights pointing at the connection between nutrition, environmental impact, sustainability, and strategies for data transparency and complex data communication along supply chains. The references of the articles found were also explored for consistency. The findings indicating different aspects of complex sustainable data transparency and their successful communication strategies through “sustainable ingredients” approach is discussed.

## The Case of Agrifood Chains

The agrifood chain is characterized by global sourcing, leading to advantages such as efficiency in resource use or availability of specialized products, but also by certain hurdles such as long-distance transportation or different legislations (31, 32). In consequence, many different private standards harmonizing legislations around the world have been established and refined in the last years (33). Beside the basic requirements of food safety, these standards request more and more sustainable as well as nutritional specifications (33) which should lead in turn to a more nutritional and sustainable diet.

The international food standards show the increasing demand of the different stakeholders along the agrifood chain. Rapidly changing consumer demand in the food area get reflection in two main development trends, especially observable in Western countries. The first is connected with consumer demand for sustainable products (34, 35), reflected in the increasing market of sustainable food marketed as regional and local foods (36), or animal welfare and fair trade products (37, 38). Secondly, consumers are more and more focusing on a healthy diet (39–41) which can be seen for example in the increasing market of functional foods supposed to deliver a health benefit beyond the nutritional value (25, 41). Food products incorporate several credence attributes relying on connections to health benefits, organic production, fairness of production and trade, sustainability, etc. (42). Consumers as well as other stakeholders along the agrifood chain are not able to control or trace all the product characteristics, for example the inherent ingredients, and so they must trust the suppliers upstream the agrifood chain and rely on any information accompanying the product and ingredients information. Examples of credence attributes are organic foods or GMO free foods (43). A good buyer-supplier relationship helps to overcome the hurdles of mistrust leading to transparency of the agrifood chain. Based on intensive buyer-supplier relationships, also aspects of a sustainable production process might be communicated through the whole chain—in best case to the very end of the chain, thus the end-consumer. The agrifood chain is often discussed to be mostly cost-driven. That means in terms of sourcing different ingredients, the price seems to be a relevant determinant (44). However, if nutritional as well as sustainable aspects should be included, there are many more aspects such as GHG emissions, water use or balanced nutritional composition to be considered during the sourcing of ingredients then the price.

Tackling the challenges around these characteristics in agrifood chains, some multinational companies already try to address them in their communication strategy toward consumers. In doing so, sustainable sourcing strategies are communicated, for example Nestlé advertises their “responsible sourcing” of their raw materials^1^ or Unilever using “sustainable sourcing” as a keyword to indicate the inclusion of sustainability aspect in supply of ingredients^2^. And as indicated before, consumers and supply chain stakeholders rely on such information as there is no option to control it. Small companies such as Impossible Foods Inc. (Redwood City, CA, USA) and Beyond Meat (El Segundo, CA, USA), producing intermediates for burger alternatives target sustainability as a crucial part of their communication and branding strategy, completely rely on aspects of lower environmental impact and health benefits as key determinants of ingredient branding. This increasing interest can be also shown in growing research on sustainable procurement in recent years (45). The basis for the communication campaigns about sustainable sourcing are mostly the description of the origin of the inherent ingredients. This can be argued as precursor for an ingredient branding strategy.

## Nutritional and Sustainable Sourcing Resulting in Ingredient Branding Strategy

### Food Credence Attributes

In the area of consumer goods—such as foods—product branding is widely discussed as key success factor (46).Ingredient branding strategy implies a brand policy for a special ingredient within the final product which is an important and sometimes a key component but cannot be clearly recognized by the user (47, 48). Thereby, the branded ingredient transfers its positive associations to the final product which enhances the perceived value of the whole product (46, 48). One prominent example of ingredient branding within the food industry is the sweetener NutraSweet^®^ which is labeled as branded ingredient on various food products (49). Another food related example would be TetraPak^®^, which found its niche as branded packaging supplier for food producers (50).

The question arises whether an ingredient branding strategy might be useful for marketing of the inherent sustainable and nutritional characteristics of a food product. As both characteristics cannot be recognized directly by consumers while consuming the product, they should be communicated via recognizable brand (48, 51). In general, there are several possibilities of building food component brands for instance by advertising campaigns using a protected trademark specifying manufacturing technologies or health benefits (52). Based on the inherent ingredients which can deliver on the one hand health benefits and on the other hand sustainable production processes an ingredient branding strategy could deliver various benefits.

The following paragraphs give an overview of the above selected characteristics of agrifood chains which result in different challenges connected to nutritional and sustainable characteristics of foods. Furthermore, first ideas how an ingredient strategy might tackle those challenges are given and briefly discussed in the following. This compilation should be further extended based on upcoming research results and does not claim to be complete.

### Globalization of Food

The globalization of agrifood chains can on the one hand deliver a better compilation of ingredients in food products which benefit human health based on the global availability of different ingredients. On the other hand, the global sourcing can lead to longer transportation which might affect the ecological outcome (53). Long transportation might also negatively affect the quality of the food due to loss of nutrients and thus negatively affect the compilation of the inherent ingredients (54). However, the global logistic system is very efficient and reliable. The consideration of these advantages and drawbacks and reliance on already developed ingredient brands with sustainable and nutritional properties creates unique opportunities for the enhancement of transparent sourcing strategy (55). Transparency in this case is an additional benefit of using ingredients with allocated sustainability attributes.

### Consumer Demand and Acceptance

Increasing consumer demands focus on healthiness of foods concurrently combined with the desire of a clear conscience in terms of sustainable shopping (56). Thus, the inherent ingredients delivering a health benefit beyond the nutritional value as well as the processing of these ingredients should be considered following nutritional as well as sustainable food characteristics. If stakeholders along the agrifood chain follow an ingredient sourcing strategy reflecting nutritional as well as sustainable aspects, final food products can be branded stating their nutritional sustainability inside. Due to the underlying transparency along the chain, this additional benefit can be also communicated toward consumers (48, 57). As the characteristics of nutritional and sustainable food ingredients cannot be recognized by different stakeholders (including the consumer) along the agrifood chain this communication might overcome mistrust.

### Food Price

The association of the branded ingredient to the final product delivers value to different stakeholders along the value chain (57, 58). It brings benefits for the supplier initiating the ingredient branding strategy in terms of higher price for the raw materials resulting in a higher revenue. The manufacturer using the ingredient can also achieve higher prices of his/her product. The retailer selling the final product can market his product with higher value to consumers and finally the consumers can benefit from the added value (for example nutritional and sustainable value) in the final product (47). It is necessary to point out here, that successful “nutritional sustainability inside” ingredient branding would depend on consumer willingness-to pay (WTP) (59). And on the other hand, consumer WTP can be triggered with level of marketing and branding of the product (including ingredient branding). Thus, along the agrifood chain different drivers of an ingredient strategy might benefit from an ingredient branding strategy.

As a sustainable supply chain management will lead to higher costs for example due to coordination effort (4), the communication strategy has to be transparent and efficient in order to induce a higher WTP to compensate higher costs (57, 60, 61). Transparency of ingredients branding “nutritional sustainability” is therefore another key credence attribute, which should be supplied and communicated together with the ingredients. The development of an efficient communication strategy, which includes stakeholders of the value chains from ingredient producers to consumers, should be set as a priority of ingredient supply companies.

### Communication Strategy

**Figure 1:**
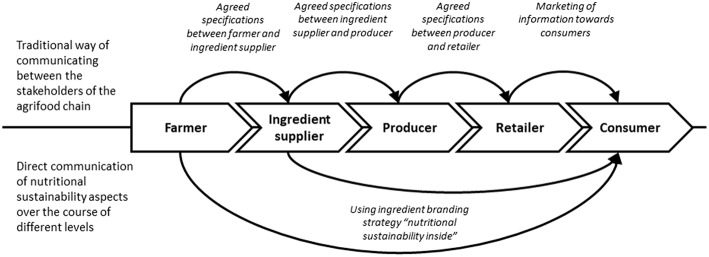
Main ways of communicating nutritional sustainability aspects along the agrifood chain.

Successful examples of emerging food ingredient companies (e.g. Impossible Foods or Beyond Meat) demonstrated that ingredient branding can be a successful communication strategy to reach consumers in Western countries and directly communicate potential nutritional or health benefits (48, 51, 52). Rising awareness of the end consumer about the benefits of branded ingredient creates preconditions for the acceptance of foods with the ingredient, higher WTP and improved communication of the brand along the supply chain (60, 62). While developed strategies for package labeling (traffic-light, nutri-score, etc.) might be a feasible basis for general communication strategies (63–65), they are not quite suitable for ingredient branding, as they are not promoting benefits of specific brand, and therefore might be less attractive for a specific producer. A direct marketing of nutritional and sustainable aspects using an ingredient branding strategy right from the front end of the agrifood chain can overcome the loss of information along the chain. This might lead to an efficient information management in which brands can market their inherent characteristics such as “nutritional sustainability inside” and grow to strong brands (ref. Figure 1).

In order to reach a final consumer with ingredient branding through a few players in the chain it is necessary to target the most important buying factors, related to food. Those are well studied and known: price, quality (health impact) and regionality (66). While nutritional sustainability is not dealing directly with the price issues, it can relate to health of people and health of the environment in a single concept transferrable between the ingredient supplier and food producers to the end consumer.

In summary, stakeholders along the agrifood chain are not able to recognize nutritional sustainability if they do not get the information from the other stakeholders as nutritional as well as sustainable characteristics cannot be checked only based on the product itself. An ingredient branding strategy relying on an appropriate sourcing and communication strategy will consequently lead to transparency along the agrifood chain if information flows from the front end of the chain to the consumers. This might lead to more nutritionally sustainable products, increasing trust among the stakeholders and a higher willingness-to-pay.

## Conclusion and Outlook

General (legal) requirements of food safety along the agrifood chain require to supply the consumers with detailed information about the raw materials and their processing in many countries around the world (see for instance European food legislation). Such requirements and data can be a starting point for informing the stakeholders about nutritional as well as sustainable characteristics of the food. Beyond that, the question arises what is necessary for a successful “nutritional sustainability inside” concept as a communication strategy.

First, the information about nutritional and sustainable characteristics of the food product is necessary enhancing transparency along the agrifood chain. Furthermore, this information must be based on beforehand defined criteria for nutritional sustainability allowing an equivalent measurement system for all companies and stakeholders along the agrifood chain. This is defined as one of the most discussed issue in recent literature about nutritional sustainability—how to measure nutritional sustainability (15, 67). Therefore, one of the main conditions for “nutritional sustainability inside” branding-based communication strategy would be the development of transparent measurement system. Second, the complexity of nutritional sustainability should be reduced to a simplified outcome based on a common measurement model, this outcome could be easily communicated throughout the whole agrifood chain for example with a label or a trademark. As findings from previous studies show that ingredient branding can successfully introduce a new attribute to the final product (68), there might be the opportunity to successfully communicate nutritional sustainability via an ingredient branding strategy. Third condition relates to the attribution of nutritional sustainability to a specific brand name. Only specific tailored brand can be effectively marketed together with defining characteristics. Specific and recognizable brand might outweigh the barriers of sustainable supply chain management such as the correlated costs.

## Author Contributions

SB, SS, and VH contributed conception and design of the study. All authors contributed to manuscript revision, read and approved the submitted version.

### Conflict of Interest Statement

SB was employed part-time by the University of Applied Sciences Osnabrück and part-time by German Institute of Food Technologies. SS and VH were employed by German Institute of Food Technologies.

## Footnotes

^1^https://www.nestle.com/csv/raw-materials

^2^https://www.unilever.com/sustainable-living/reducing-environmental-impact/sustainable-sourcing/
